# Apocynin influence on oxidative stress and cardiac remodeling of spontaneously hypertensive rats with diabetes mellitus

**DOI:** 10.1186/s12933-016-0442-1

**Published:** 2016-09-01

**Authors:** C. M. Rosa, R. Gimenes, D. H. S. Campos, G. N. Guirado, C. Gimenes, A. A. H. Fernandes, A. C. Cicogna, R. M. Queiroz, I. Falcão-Pires, D. Miranda-Silva, P. Rodrigues, F. R. Laurindo, D. C. Fernandes, C. R. Correa, M. P. Okoshi, K. Okoshi

**Affiliations:** 1 Department of Internal Medicine, Botucatu Medical School, Sao Paulo State University-UNESP, Botucatu, Sao Paulo Brazil; 2 Sagrado Coração University-USC, Bauru, Sao Paulo Brazil; 3 Department of Chemistry and Biochemistry, Institute of Biosciences, Sao Paulo State University-UNESP, Botucatu, Sao Paulo Brazil; 4 Department of Physiology and Cardiothoracic Surgery, Faculty of Medicine, University of Porto, Porto, Portugal; 5 Department of Cardiopneumology, Medical School, Sao Paulo University-USP, Sao Paulo, Brazil; 6 Department of Pathology, Medical School, Sao Paulo State University-UNESP, Botucatu, Sao Paulo Brazil

**Keywords:** Systemic hypertension, Ventricular remodeling, Oxidative stress, NADPH oxidase, Myocardial fibrosis, Spontaneously hypertensive rat

## Abstract

**Purpose:**

Although increased oxidative stress is a major component of diabetic hypertensive cardiomyopathy, research into the effects of antioxidants on cardiac remodeling remains scarce. The actions of antioxidant apocynin include inhibiting reactive oxygen species (ROS) generation by nicotinamide adenine dinucleotide phosphate (NADPH) oxidases and ROS scavenging. We evaluated the effects of apocynin on cardiac remodeling in spontaneously hypertensive rats (SHR) with diabetes mellitus (DM).

**Methods:**

Male SHR were divided into four groups: control (SHR, n = 16); SHR treated with apocynin (SHR-APO; 16 mg/kg/day, added to drinking water; n = 16); diabetic SHR (SHR-DM, n = 13); and SHR-DM treated with apocynin (SHR-DM-APO, n = 14), for eight weeks. DM was induced by streptozotocin (40 mg/kg, single dose). Statistical analyzes: ANOVA and Tukey or Mann–Whitney.

**Results:**

Echocardiogram in diabetic groups showed higher left ventricular and left atrium diameters indexed for body weight, and higher isovolumetric relaxation time than normoglycemic rats; systolic function did not differ between groups. Isolated papillary muscle showed impaired contractile and relaxation function in diabetic groups. Developed tension was lower in SHR-APO than SHR. Myocardial hydroxyproline concentration was higher in SHR-DM than SHR, interstitial collagen fraction was higher in SHR-DM-APO than SHR-APO, and type III collagen protein expression was lower in SHR-DM and SHR-DM-APO than their controls. Type I collagen and lysyl oxidase expression did not differ between groups. Apocynin did not change collagen tissue. Myocardial lipid hydroperoxide concentration was higher in SHR-DM than SHR and SHR-DM-APO. Glutathione peroxidase activity was lower and catalase higher in SHR-DM than SHR. Apocynin attenuated antioxidant enzyme activity changes in SHR-DM-APO. Advanced glycation end-products and NADPH oxidase activity did not differ between groups.

**Conclusion:**

Apocynin reduces oxidative stress independently of NADPH oxidase activity and does not change ventricular or myocardial function in spontaneously hypertensive rats with diabetes mellitus. The apocynin-induced myocardial functional impairment in SHR shows that apocynin actions need to be clarified during sustained chronic pressure overload.

## Background

Systemic arterial hypertension and diabetes mellitus (DM) represent two important risk factors for cardiovascular disease, the leading cause of morbidity and mortality in the world [1, 2]. The coexistence of arterial hypertension and diabetes is commonly observed in adults and has been associated with a higher risk of cardiovascular events [1–3]. In the heart, the combination of DM and hypertension, also known as diabetic hypertensive cardiomyopathy leads to more extensive structural and functional cardiac changes than either condition alone [4–6]. However, the physiopathology of diabetes and hypertension-induced cardiac disease is not completely clear. Diabetes in hypertensive rodents impairs cardiac remodeling by exacerbating fibrosis, inflammation, microvascular changes, and oxidative stress resulting in intensified myocardial and cardiac dysfunction and increased mortality rate [5–11].

One of the major components of diabetic hypertensive cardiomyopathy is an increase in oxidative stress [5, 12, 13]. The term oxidative stress refers to disturbance in redox hemostasis occurring when reactive oxygen species (ROS) production exceeds their degradation by anti-oxidant defenses [14]. In diabetes, oxidative stress can be induced by hyperglycemia, hyperlipidemia, and inflammation. Antioxidants have been extensively evaluated for their potential to prevent or treat diabetic complications [15, 16]; however, research into the effects of anti-oxidants on cardiac remodeling remains scarce [17, 18].

Apocynin, a constituent of root extracts of the medicinal herb *Picrorhiza kurroa* [19], has been used in experimental studies as an antioxidant agent. Its actions include inhibiting ROS generation by the nicotinamide adenine dinucleotide phosphate (NADPH) oxidases and ROS scavenging [14, 20, 21]. NADPH oxidases produce ROS as their primary function and can be involved in the pathophysiology of cardiac diseases [14, 22–24]. As hyperglycemia increases NADPH oxidase activity [23–25], administration of its inhibitors has been tested in diabetic cardiomiopathy [26–29]. Studies performed in rodents with streptozotocin-induced DM have shown that NADPH oxidase activity is increased in DM [30]. Furthermore, inhibition of NADPH oxidase by apocynin alleviated myocardial contractile dysfunction in DM [28, 31, 32]. However, few studies have analyzed the effects of apocynin in diabetic and hypertensive animals [33], and none in cardiac remodeling. Despite the potential benefits of apocynin in oxidative stress, its effects are not completely understood and even pro-oxidant action has been reported in some experimental models [34, 35]. In this study, we evaluated the influence of apocynin on cardiac remodeling in spontaneously hypertensive rats with diabetes mellitus.

## Methods

### Experimental groups

Seven-month-old male spontaneously hypertensive rats (SHR) were purchased from the Multidisciplinary Center for Biological Investigation in Laboratory Animals Science, State University of Campinas, SP, Brazil. All animals were housed in a room under temperature control at 23 °C and kept on a 12-h light/dark cycle. Commercial chow and water were supplied ad libitum. The rats were assigned into four groups: control SHR (n = 16); SHR treated with apocynin (SHR-APO, n = 16); diabetic SHR (SHR-DM, n = 18); and diabetic SHR treated with apocynin (SHR-DM-APO, n = 19).

Diabetes was induced by intraperitoneal injection of streptozotocin (Sigma, St. Louis, MO, USA) at 40 mg/kg body weight diluted in 0.01 M citrate buffer pH 4.5 [36]. Control groups received an intraperitoneal injection of vehicle only. As the rats received only one moderate dose of streptozotocin, sucrose administration was not necessary to avoid hypoglycemia caused by sudden release of insulin due to massive islet β-cells necrosis [37]. Seven days after streptozotocin administration, blood glucose was measured by glucometer (Advantage®). Only rats with glycemia >220 mg/dL were considered diabetic and included in the study [5, 36]. Apocynin (Sigma, St. Louis, MO, USA) was added to drinking water at a dosage of 16 mg/kg/day for 8 weeks [38]. Water consumption was measured daily and body weight weekly. Systolic arterial pressure was measured before streptozotocin injection and at the end of experiment by tail-cuff method using a model 709-0610 electro-sphygmomanometer (*Narco Bio*-*System*^®^, *International Biomedical Inc*., USA).

### Echocardiographic study

Echocardiographic evaluation was performed before diabetes induction and at the end of the experimental period. We used a commercially available echocardiograph (General Electric Medical Systems, Vivid S6, Tirat Carmel, Israel) equipped with a 5–11.5 MHz multifrequency probe, as previously described [39–41]. Rats were anesthetized by intramuscular injection of a mixture of ketamine (50 mg/kg) and xylazine (0.5 mg/kg). Two-dimensionally guided M-mode images were obtained from short-axis views of the left ventricle (LV) just below the tip of the mitral-valve leaflets, and at the level of the aortic valve and left atrium. M-mode images were printed on a thermal printer (Sony UP-890MD) at a sweep speed of 100 mm/s. All LV structures were manually measured by the same observer (KO). Values obtained were the mean of at least five cardiac cycles on M-mode tracings. The following structural variables were measured: left atrium diameter (LA), LV diastolic diameter (LVDD), and LV diastolic and systolic posterior wall thickness (PWT and SWT, respectively). LV relative wall thickness (RWT) was calculated using the formula 2 × PWT/LVDD. LV mass was calculated using the formula [(LVDD + PWT + SWT)^3^ − (LVDD)^3^] × 1.04. LV function was assessed by the following parameters: midwall fractional shortening (MFS), tissue Doppler imaging (TDI) of mitral annulus systolic velocity (S’), myocardial performance index (Tei index), early and late diastolic mitral inflow velocities (E and A waves), E/A ratio, isovolumetric relaxation time (IVRT), TDI of early mitral annulus diastolic velocity (E’), and E/E’ ratio. Tissue Doppler variables (S’ and E’) were measured at the septal and lateral walls, and average values presented [42].

### Myocardial functional evaluation

At the end of the experimental period, myocardial contractile performance was evaluated in isolated LV papillary muscle preparation as previously described [43, 44]. Rats were anesthetized (pentobarbital sodium, 50 mg/kg, intraperitoneally) and decapitated. Hearts were quickly removed and placed in oxygenated Krebs–Henseleit solution at 28 °C. LV anterior or posterior papillary muscle was dissected free, mounted between two spring clips, and placed vertically in a chamber containing Krebs–Henseleit solution at 28 °C and oxygenated with a mixture of 95 % O_2_ and 5 % CO_2_ (pH 7.38). The composition of the Krebs–Henseleit solution in mM was as follows: 118.5 NaCl, 4.69 KCl, 1.25 CaCl_2_, 1.16 MgSO_4_, 1.18 KH_2_PO_4_, 5.50 glucose, and 25.88 NaHCO_3_. The spring clips were attached to a Kyowa model 120T-20B force transducer and a lever system, which allowed for muscle length adjustment. Preparations were stimulated 12 times/min at a voltage 10 % above threshold.

After a 60 min period, during which the preparations were permitted to shorten while carrying light loads, muscles were loaded to contract isometrically and stretched to the apices of their length-tension curves. After a 5 min period, during which preparations performed isotonic contractions, muscles were again placed under isometric conditions, and the apex of the length-tension curve (L_max_) was determined. A 15 min period of stable isometric contraction was imposed prior to the experimental period. One isometric contraction was then recorded for later analysis.

The following parameters were measured from the isometric contraction: peak developed tension (DT), resting tension (RT), time to peak tension (TPT), maximum tension development rate (+dT/dt), and maximum tension decline rate (−dT/dt). To evaluate myocardial contractile reserve, papillary muscle mechanical performance was evaluated after the following positive inotropic stimulation: 30 s post-rest contraction, extracellular Ca^2+^ concentration increase to 2.5 mM, and β-adrenergic agonist isoproterenol (10^−6^ M) addition to the nutrient solution [45]. Papillary muscle cross-sectional area (CSA) was calculated from muscle weight and length by assuming cylindrical uniformity and a specific gravity of 1.0. All force data were normalized for muscle CSA.

### Histologic analysis

Transverse LV sections were fixed in 10 % buffered formalin and embedded in paraffin. Sections (5 µm thick) were stained with hematoxylin–eosin and collagen-specific stain picrosirius red (Sirius red F3BA in aqueous saturated picric acid) [46]. In at least 50 myocytes from each LV, where the nucleus could clearly be identified, the smallest transverse diameters were measured [47]. On average, 20 microscopic fields were used to quantify interstitial collagen fraction. Perivascular collagen was excluded from this analysis [48]. Measurements were performed using a Leica microscope (magnification 40×) attached to a video camera and connected to a computer equipped with image analysis software (Image-Pro Plus 3.0, Media Cybernetics, Silver Spring, MD, USA).

### Myocardial hydroxyproline concentration

Hydroxyproline concentration was measured in LV to estimate myocardial collagen content [49]. Tissue was dried using a Speedvac Concentrator (SC 100) attached to a refrigerated condensation trap (TRL 100) and vacuum pump (VP 100, Savant Instruments, Inc., Farmingdale, NY, USA). Dry tissue weight was measured and samples were hydrolyzed overnight at 100 °C with 6 N HCl (1 mL/10 mg dry tissue). An aliquot of the hydrolysate (50 μL) was transferred to an eppendorf tube and dried in the speedvac concentrator. One milliliter of deionized water was added and the sample transferred to a tube with a teflon screw cap. One milliliter of potassium borate buffer (pH 8.7) was added to maintain constant pH and the sample was oxidized with 0.3 mL of chloramine T solution at room temperature for 20 min. The addition of 1 mL of 3.6 M/L sodium thiosulfate with thorough mixing for 10 s stopped the oxidative process. The solution was saturated with 1.5 g KCl. The tubes were heated in boiling water for 20 min. After cooling to room temperature, the aqueous layer was extracted with 2.5 mL of toluene. One and a half milliliters of toluene extract were transferred to a 12 × 75 mm test tube. Then 0.6 mL of Ehrlich’s reagent was added and color allowed to develop for 30 min. Absorbance was read at 565 nm against a reagent blank. Deionized water and 20 μg/mL hydroxyproline were used as the blank and standard, respectively.

### Western blotting analysis

Protein expression of type I (Santa Cruz Biotechnology Inc., Santa Cruz, CA, USA, col1a1, sc-8784-r) and type III collagen (Abcam, Cambridge, UK, col3a1, ab6310) and lysil oxidase (Abcam, LOX1, ab60178) was analyzed by Western blot as previously described [50, 51]. Samples were separated on polyacrylamide gel and then transferred to a nitrocellulose membrane. After blockage, the membrane was incubated with the primary antibodies overnight at 4 °C. The membrane was then washed with PBS and Tween 20 and incubated with secondary peroxidase-conjugated antibodies (Santa Cuz Biotechnology, anti-mouse, sc-2005, and anti-rabbit, sc-2004) for 90 min at room temperature. ECL western blotting substrate (Pierce Protein Research Products, Rockford, USA) was used to detect bound antibodies. The membrane was then stripped (Restore Western Blot Stripping Buffer, Pierce Protein Research Products, Rockford, USA) to remove antibodies. After blockage, membrane was incubated with anti-GAPDH antibody (Santa Cruz Biotechnology, GAPDH 6C5 sc-32233). Protein levels were normalized to GAPDH.

### Myocardial oxidative stress evaluation

Frozen left ventricle myocardium (∼200 mg) was homogenized in phosphate buffer (0.1 M) pH 7.4 and centrifuged at 12,000*g* for 15 min at 4 °C. The supernatant was assayed for total protein, lipid hydroperoxide, and anti-oxidant enzyme activity [52]. Lipid hydroperoxide concentration was determined in a medium containing methanol 90 % (v/v), 250 μM ammonium ferrous sulfate, 100 μM xylenol orange, 25 mM sulfuric acid, and 4 mM butylated hydroxytoluene. The solution was incubated for 30 min at room temperature and measurement was performed at 560 nm. Glutathione peroxidase (GSH-Px, E.C.1.11.1.9) was assayed using 0.15 M phosphate buffer, pH 7.0, containing 5 mM EDTA, 0.1 mL of 0.0084 M NADPH, 4 μg of GSSG-reductase, 1.125 M sodium azide, and 0.15 M glutathione reduced form (GSH) in a total volume of 0.3 mL. Superoxide dismutase (SOD, E.C.1.15.1.1.) activity was determined based on its ability to inhibit reduction of nitroblue tetrazolium, in a medium containing 50 mM phosphate buffer pH 7.4, 0.1 mM EDTA, 50 μM nitroblue tetrazolium, 78 μM NADH, and 3.3 μM phenazine methosulfate. One unit of SOD was defined as the amount of protein needed to decrease the reference rate to 50 % of maximum inhibition. Catalase (E.C.1.11.1.6.) activity was evaluated in 50 mM phosphate buffer, pH 7.0, with 10 mM hydrogen peroxide. One unit of catalase was defined as the amount of enzyme needed to degrade 1 μmol H_2_O_2_ over 60 s, at 240 nm. Enzyme activity was analyzed at 25 °C using a microplate reader system (μQuant-MQX 200 with KC Junior software, Bio-TeK Instruments, Winooski, VT, USA). All reagents were purchased from Sigma (St. Louis, MO, USA).

### Advanced glycation end-products (AGE) quantification

Paraffin-embedded myocardial tissue was cut, mounted on slides for immunohistochemical analysis, and deparaffinized with EDTA for antigen retrieval [53, 54]. Blockade was performed with hydrogen peroxide, goat serum, and avidin/biotin. Tissue sections were incubated with primary antibody (rabbit polyclonal antibody to AGE; Ab23722, Abcam) for 1 h, then with a secondary antibody (goat polyclonal secondary antibody to rabbit IgG HL—Biotin; Ab6720, Abcam) for 2 h, at room temperature, and for 15 min with streptavidin. Sections were then stained with 3,3′-diaminobenzidine and counterstained with hematoxylin [53]. Analysis was performed using a video camera coupled to a microscope connected to a computer with an image analyzes program (Image Pro Plus 6.0, Media Cybernetics, Silver Spring, Maryland, USA). Cardiac tissue components were identified according to color enhancement. AGEs yield a brown color while myocytes are seen in purple. Areas containing blood vessels were excluded from this analysis.

### NADPH oxidase activity

NADPH oxidase activity was evaluated in membrane-enriched cellular fraction by quantifying dihydroethidium (DHE) oxidation-derived fluorescent compounds, 2-hydroxyethidium (EOH) and ethidium, by HPLC according to a previously described method [55, 56]. Cardiac muscle was washed in PBS to remove blood. Muscle fragments (∼200 mg) were homogenized in 1 mL of ice-cold lysis buffer containing 50 mM Tris (pH 7.4), 100 mM DTPA, 0.1 % β-mercaptoethanol, and protease inhibitors. The samples were then sonicated (3 cycles of 10 s at 8 W) and centrifuged at 1000*g* for 3 min, at 4 °C. The supernatant was transferred to another microtube and centrifuged at 18,000*g* for 10 min at 4 °C. The supernatant was then centrifuged at 100,000*g* for 45 min, at 4 °C. The supernatant was discarded and the pellet resuspended in 100 µL of lysis buffer [56]. Total protein content was quantified by the Bradford method. Subsequently, 20 µg of membrane-enriched cellular fraction was incubated in phosphate buffer (50 mM, pH 7.4, with 0.1 mM DTPA) containing DHE (50 µM) and NADPH (300 µM), to a final volume of 100 µL, for 30 min at 37 °C in the dark. After adding 40 µL of 10 % trichloroacetic acid, the samples were ice-cooled for 10 min in the dark, and centrifuged at 12,000*g* for 10 min at 4 °C. The supernatant was analyzed by HPLC and the fluorescent DHE-derived products were quantified, as previously described [55, 56].

### Statistical analysis

Results are expressed as mean and standard deviation or median and 25th and 75th percentiles according to normal or non-normal distribution, respectively. Variables were compared by two-factor ANOVA followed by the Tukey test for normal distribution data or the Dunn test for non-normal distribution data. All rats were subjected to functional studies as their parameters usually present an elevated variability. In papillary muscle preparations, some animals were discarded due to technical problems. For all morphological and molecular analyzes, samples were randomly chosen. Statistical significance was accepted at p < 0.05.

## Results

Initial and final body weight, heart rate, systolic blood pressure, and blood glucose are shown in Table 1. Among streptozotocin injected rats, 27.5 % did not develop diabetes, and were excluded from the study. At the end of protocol both diabetic groups presented lower body weight and higher glycemia compared to the respective control groups; apocynin administration did not change body weight, heart rate or glycemia. Initial and final blood pressure did not differ between groups.

**Table 1:** Initial and final body weight, heart rate, systolic blood pressure, and blood glucose

	SHR (n = 16)	SHR-APO (n = 16)	SHR-DM (n = 13)	SHR-DM-APO (n = 14)
Initial BW (g)	360 ± 27	361 ± 28	356 ± 16	359 ± 18
Final BW (g)	362 ± 25	364 ± 27	254 ± 36*	239 ± 37^#^
Initial heart rate (bpm)	294 ± 40.4	304 ± 46.7	308 ± 50.8	304 ± 46.7
Final heart rate (bpm)	269 ± 48.3	287 ± 45.0	255 ± 38.5	249 ± 35.9^#^
Initial BP (mm Hg)	189 ± 19	189 ± 18	191 ± 18	190 ± 17
Final BP (mm Hg)	184 ± 22	186 ± 21	187 ± 28	184 ± 25
Initial glucose mg/dL)	90 (83–101)	93 (83–100)	91 (90–100)	94 (90–99)
Final glucose (mg/dL)	93 (88–101)	94 (88–107)	600 (542–600)*	600 (538–600)^#^

Data are expressed as mean ± standard deviation or median and 25th and 75th percentiles

*SHR* spontaneously hypertensive rats; *SHR*-*APO* SHR treated with apocynin; *SHR*-*DM* diabetic SHR; *SHR*-*DM*-*APO* diabetic SHR treated with apocynin; *BW* body weight; *bpm* beats per min; *BP* systolic blood pressure

Two-factor ANOVA; * p < 0.05 vs SHR; ^#^ p < 0.05 vs SHR-APO

At the beginning of the protocol, all echocardiographic variables were similar between groups (data not shown). Echocardiographic structural data assessed at the end of the protocol (Table 2) showed higher normalized LV diastolic diameter and left atrium diameter in both diabetic compared to normoglycemic groups. LV mass-to-body weight ratio was higher in SHR-DM than SHR, and LV posterior wall thickness was lower in SHR-DM-APO than SHR-APO. LV functional parameters are presented in Table 3. Systolic function did not differ between groups. Isovolumetric relaxation time was higher in diabetic groups, indicating diastolic dysfunction.

**Table 2:** Echocardiographic structural data

	SHR (n = 16)	SHR-APO (n = 16)	SHR-DM (n = 13)	SHR-DM-APO (n = 14)
LVDD/BW (mm/kg)	19.5 ± 1.87	20.4 ± 2.27	26.2 ± 5.04*	26.6 ± 4.71^#^
LA/BW (mm/kg)	17.3 ± 2.06	17.3 ± 2.14	20.5 ± 3.97*	21.1 ± 3.23^#^
PWT (mm)	1.69 ± 0.17	1.71 ± 0.19	1.63 ± 0.17	1.55 ± 0.16^#^
RWT	0.46 ± 0.06	0.45 ± 0.07	0.47 ± 0.07	0.46 ± 0.07
LVM/BW (g/kg)	2.34 ± 0.37	2.54 ± 0.57	2.89 ± 0.64*	2.70 ± 0.59

Data are expressed as mean ± standard deviation

*SHR* spontaneously hypertensive rats; *SHR*-*APO* SHR treated with apocynin; *SHR*-*DM* diabetic SHR; *SHR*-*DM*-*APO* diabetic SHR treated with apocynin; *LVDD* left ventricular (LV) diastolic diameter; *BW* body weight; *LA* left atrial diameter; *PWT* LV posterior wall thickness; *RWT* relative wall thickness; *LVM* LV mass

Two-factor ANOVA; * p < 0.05 vs SHR; ^#^ p < 0.05 vs SHR-APO

**Table 3:** Echocardiographic left ventricular functional data

	SHR (n = 16)	SHR-APO (n = 16)	SHR-DM (n = 13)	SHR-DM-APO (n = 14)
MFS (%)	25.9 ± 3.02	25.1 ± 3.53	25.8 ± 2.36	26.3 ± 3.05
TDI S’ (average, cm/s)	3.25 (2.63–3.38)	2.75 (2.50–3.19)	3.00 (2.75–3.00)	2.75 (2.50–3.00)
Tei index	0.61 ± 0.09	0.61 ± 0.12	0.60 ± 0.09	0.61 ± 0.12
E/A	1.72 ± 0.56	1.64 ± 0.58	1.38 ± 0.45	1.70 ± 0.38
IVRT (ms)	31.9 ± 3.12	32.9 ± 5.05	37.2 ± 4.00*	38.9 ± 5.57^#^
E/E’	23.3 ± 4.65	24.3 ± 5.03	23.6 ± 4.19	24.1 ± 5.99

Data are expressed as mean ± standard deviation or median and 25th and 75th percentiles

*SHR* spontaneously hypertensive rats; *SHR*-*APO* SHR treated with apocynin; *SHR*-*DM* diabetic SHR; *SHR*-*DM*-*APO* diabetic SHR treated with apocynin; *MFS* midwall fractional shortening; *TDI S’* tissue Doppler imaging (TDI) of mitral annulus systolic velocity; *E/A* ratio between early (E)-to-late (A) diastolic mitral inflow; *IVRT* isovolumic relaxation time; *E’* TDI of early mitral annulus diastolic velocity

Two-factor ANOVA; * p < 0.05 vs SHR; ^#^ p < 0.05 vs SHR-APO

Data from LV papillary muscle function at basal condition are shown in Table 4. Apocynin decreased developed tension only in non-diabetic rats. The +dT/dt and −dT/dt were lower and time to peak tension higher in both diabetic groups independently of apocynin treatment. Results from positive inotropic stimulation are shown in Table 5. In 30 s post-rest contraction, developed tension and +dT/dt were lower in SHR-APO than SHR. After all inotropic stimulation, −dT/dt was lower and time to peak tension higher in both diabetic groups than their respective controls, and +dT/dt was lower in SHR-DM than SHR. SHR-DM-APO did not differ from SHR-DM, except by −dT/dt which was lower in SHR-DM-APO during 30 s post-rest contraction.

**Table 4:** LV papillary muscle data

	SHR (n = 13)	SHR-APO (n = 11)	SHR-DM (n = 11)	SHR-DM-APO (n = 10)
DT (g/mm^2^)	11.2 ± 2.37	9.22 ± 1.35*	10.5 ± 2.65	9.17 ± 2.52
RT (g/mm^2^)	1.06 ± 0.29	1.09 ± 0.30	1.10 ± 0.40	1.11 ± 0.63
+dT/dt (g/mm^2^/s)	112 ± 24.9	96.2 ± 18.2	92.4 ± 23.1*	85.0 ± 24.2^#^
TPT (ms)	203 ± 18.4	189 ± 14.5	235 ± 18.6*	227 ± 17.7^#^
−dT/dt (g/mm^2^/s)	40.6 ± 10.1	35.1 ± 5.05	33.3 ± 8.92*	27.5 ± 8.31^#^
CSA (mm^2^)	0.71 ± 0.13	0.78 ± 0,12	0.72 ± 0.11	0.75 ± 0.14

Data are expressed as mean ± standard deviation

*SHR* spontaneously hypertensive rats; *SHR*-*APO* SHR treated with apocynin; *SHR*-*DM* diabetic SHR; *SHR*-*DM*-*APO* diabetic SHR treated with apocynin; *DT* peak of developed tension; *RT* resting tension; +*dT/dt* maximum rate of tension development; *TPT* time to peak tension; −*dT/dt* maximum rate of tension decline; *CSA* papillary muscle cross sectional area

Two-factor ANOVA; * p < 0.05 vs SHR; ^#^ p < 0.05 vs SHR-APO

**Table 5:** Isolated papillary muscle data after positive inotropic stimulation

		SHR (n = 13)	SHR-APO (n = 11)	SHR-DM (n = 11)	SHR-DM-APO (n = 10)
PP30	DT	13.7 ± 3.09	11.1 ± 1.69*	11.9 ± 3.01	10.3 ± 2.87
	RT	1.04 ± 0.30	1.06 ± 0.31	1.24 ± 0.52	1.25 ± 0.62
	+dT/dt	140 ± 33.4	115 ± 23.1*	105 ± 25.0*	95.2 ± 27.8
	TPT	215 ± 17.1	209 ± 13.0	255 ± 27.0*	250 ± 22.6^#^
	−dT/dt	42.6 ± 9.67	35.8 ± 4.58	32.4 ± 8.73*	25.1 ± 8.45^#§^
2.5 mM [Ca^+2^]_0_	DT	12.5 ± 2.78	10.3 ± 1.49	11.0 ± 2.95	9.79 ± 2.85
	RT	0.87 ± 0.30	0.90 ± 0.31	0.90 ± 0.34	0.92 ± 0.52
	+dT/dt	140 ± 31.9	117 ± 21.4	108 ± 28.0*	97.9 ± 28.7
	TPT	192 ± 16.9	186 ± 17.8	224 ± 23.2*	216 ± 15.1^#^
	−dT/dt	48.3 ± 10.6	40.0 ± 6.27	38.4 ± 11.6*	30.7 ± 10.2^#^
Iso 10^−6^ M	DT	11.4 (8.33–13.5)	9.70 (8.77–10.1)	9.68 (8.60–10.1)	9.47 (6.08–10.8)
	RT	0.85 ± 0.43	0.86 ± 0.34	0.84 ± 0.28	0.83 ± 0.52
	+dT/dt	136 ± 34.0	118 ± 22.4	96.9 ± 26.6*	96.7 ± 31.7
	TPT	163 ± 11.1	157 ± 9.49	184 ± 16.9*	190 ± 15.8^#^
	−dT/dt	67.9 ± 16.6	58.3 ± 8.03	46.4 ± 13.4*	39.1 ± 13.7^#^

Data are expressed as mean ± standard deviation or median and 25th and 75th percentile

*SHR* spontaneously hypertensive rats; *SHR*-*APO* SHR treated with apocynin; *SHR*-*DM* diabetic SHR; *SHR*-*DM*-*APO* diabetic SHR treated with apocynin; *PP30* 30 s post-rest contraction; *2.5* *mM [Ca* ^+*2*^ *]*_*0*_ extracellular Ca^2+^ concentration increased to 2.5 mM; *Iso 10* ^−*6*^ *M* β-adrenergic agonist isoproterenol (10^−6^ M) added to the nutrient solution; *DT* peak of developed tension (g/mm^2^); *RT* resting tension (g/mm^2^); +*dT/dt* maximum rate of tension development (g/mm^2^/s); *TPT* time to peak tension (ms); −*dT/dt* maximum rate of tension decline (g/mm^2^/s)

Two-factor ANOVA; * p < 0.05 vs SHR; ^#^ p < 0.05 vs SHR-APO; ^§^ p < 0.05 vs SHR-DM

Myocyte diameter did not differ between groups (Table 6). Regarding the extracellular matrix, left ventricular myocardial hydroxyproline concentration was higher in SHR-DM than SHR and interstitial collagen fraction was higher in SHR-DM-APO than SHR-APO. Specifically, type III collagen expression was lower in both diabetic groups (Table 7).

**Table 6:** Left ventricular morphometric parameters and hydroxyproline concentration

	SHR (n = 10)	SHR-APO (n = 10)	SHR-DM (n = 10)	SHR-DM-APO (n = 10)
Diameter (μm)	11.1 (10.3–12.1)	10.3 (10.3–12.2)	10.2 (10.1–12.1)	10.6 (9.88–11.0)
ICF (%)	6.95 ± 2.57	5.47 ± 2.41	9.93 ± 3.59	9.47 ± 4.30^#^
HOP (mg/g)	2.66 ± 0.29	3.08 ± 0.58	3.27 ± 0.45*	3.29 ± 0.51

Data are expressed as mean ± standard deviation or median and 25th and 75th percentile

*SHR* spontaneously hypertensive rats; *SHR*-*APO* SHR treated with apocynin; *SHR*-*DM* diabetic SHR; *SHR*-*DM*-*APO* diabetic SHR treated with apocynin; *Diameter* myocyte lower diameter; *HOP* myocardial hydroxyproline concentration; *ICF* myocardial interstitial collagen fraction

Two-factor ANOVA; * p < 0.05 vs SHR; ^#^ p < 0.05 vs SHR-APO

**Table 7:** Left ventricular myocardial protein expression

	SHR (n = 7)	SHR-APO (n = 7)	SHR-DM (n = 7)	SHR-DM-APO (n = 7)
Type I collagen	1.00 ± 0.41	1.08 ± 0.33	0.99 ± 0.28	0.96 ± 0.14
Type III collagen	1.00 ± 0.10	0.97 ± 0.11	0.77 ± 0.17*	0.83 ± 0.10^#^
Type I/III collagen ratio	1.00 ± 0.37	1.16 ± 0.42	1.33 ± 0.33	1.15 ± 0.13
Lysyl oxidase	1.00 ± 0.19	0.91 ± 0.15	0.90 ± 0.20	0.89 ± 0.13

Data are expressed as mean ± standard deviation

*SHR* spontaneously hypertensive rats; *SHR*-*APO* SHR treated with apocynin; *SHR*-*DM* diabetic SHR; *SHR*-*DM*-*APO* diabetic SHR treated with apocynin

Two-factor ANOVA; * p < 0.05 vs SHR; ^#^ p < 0.05 vs SHR-APO

**Fig. 1:**
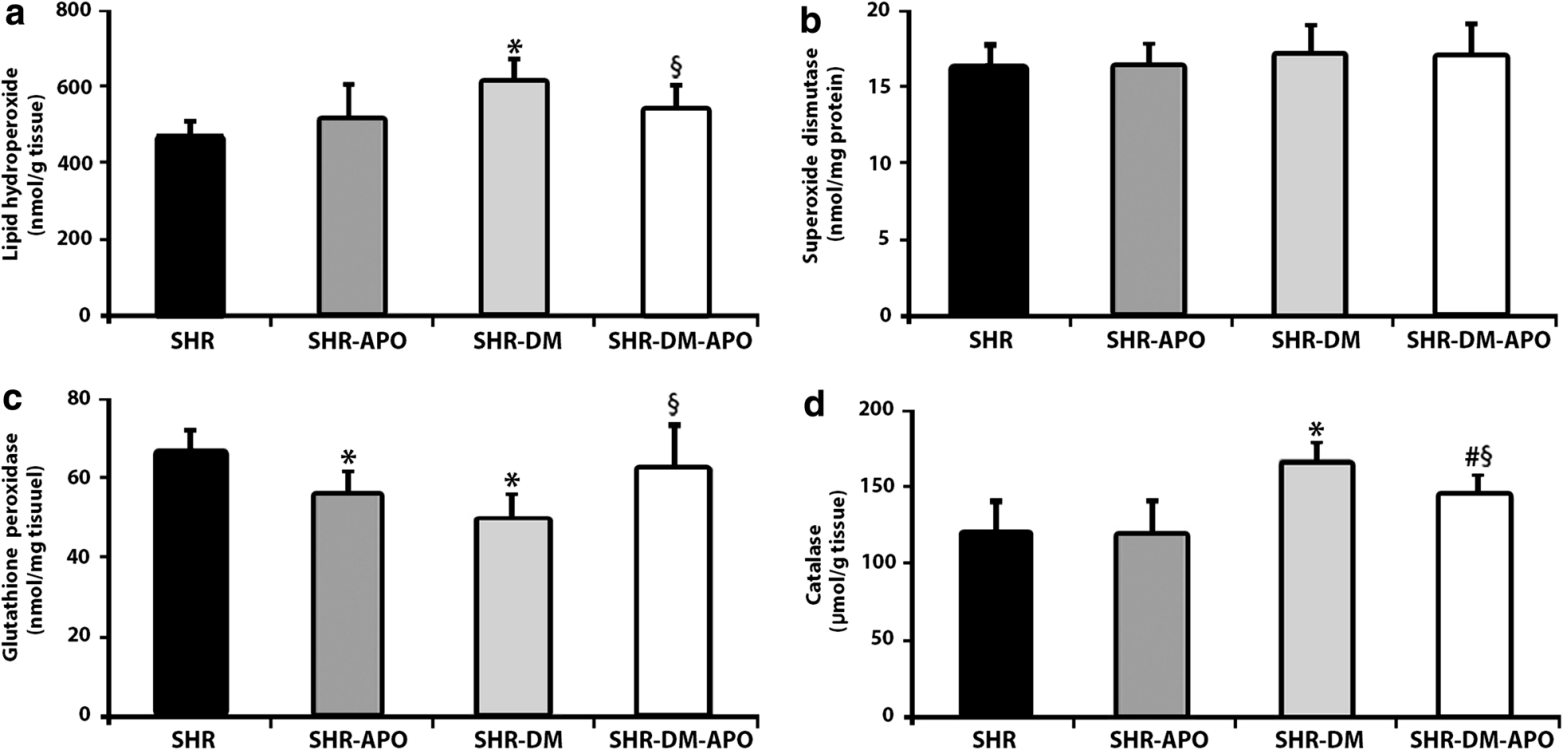
Left ventricular myocardial oxidative stress markers. Lipid hydroperoxide (**a**), superoxide dismutase (**b**), glutathione peroxidase (**c**) and catalase (**d**). Data are expressed as mean ± standard deviation. *SHR* spontaneously hypertensive rats; *SHR-APO* SHR treated with apocynin; *SHR-DM* diabetic SHR; *SHR-DM-APO* diabetic SHR treated with apocynin. Two-factor ANOVA; *p < 0.05 vs SHR; ^#^p < 0.05 vs SHR-APO; ^§^p < 0.05 vs SHR-DM-APO

Oxidative stress parameters are shown in Fig. 1. Lipid hydroperoxide concentration was higher in SHR-DM than SHR and SHR-DM-APO. Glutathione peroxidase activity was lower in SHR-APO and SHR-DM than SHR and higher in SHR-DM-APO than SHR-DM. NADPH oxidase activity and advanced glycation end-products did not differ between groups Table 8.

**Table 8:** Left ventricular myocardial oxidative stress

	SHR (n = 8)	SHR-APO (n = 8)	SHR-DM (n = 8)	SHR-DM-APO (n = 8)
AGEs	2.47 (2.35–4.16)	2.54 (1.90–3.86)	2.61 (1.83–4.16)	2.68 (2.09–4.97)
DHE	39.1 (38.2–39.7)	39.9 (39.0–40.2)	39.5 (37.4–40.6)	39.4 (38.2–40.2)
EOH	39.6 (31.9–47.6)	38.4 (31.7–40.2)	34.5 (22.4–38.0)	33.2 (29.1–40.1)
E	112 (86.6–165)	97.5 (85.9–222)	119 (112–163)	129 (106–164)

Data are expressed as mean ± standard deviation or median and 25th and 75th percentile

*SHR* spontaneously hypertensive rats; *SHR*-*APO* SHR treated with apocynin; *SHR*-*DM* diabetic SHR; *SHR*-*DM*-*APO* diabetic SHR treated with apocynin; *AGEs* advanced glycation end-products (%); *DHE* dihydroethidium (µM); *EOH* 2-hydroxyethidium (µM); *E* ethidium (µM)

Two-factor ANOVA; p > 0.05

## Discussion

In this study we evaluated the influence of anti-oxidant apocynin on cardiac remodeling in spontaneously hypertensive rats with type 1 diabetes mellitus

SHR has extensively been used to evaluate long-term pressure overload-induced cardiac remodeling [48]. At 1 month old, arterial hypertension starts to increase stimulating left ventricular hypertrophy, which remains functionally stable until 18–22 months of age [57, 58]. Therefore, in this study we evaluated seven-month-old rats as they had already developed arterial hypertension and stable left ventricular hypertrophy.

Diabetes was caused by streptozotocin, which has been widely used to induce type 1 diabetes mellitus in rodents [59–61]. Rats with streptozotocin-induced DM can develop several diabetic complications, such as neuropathy, nephropathy, and atherosclerosis [37]. The models are used not only to study pathological consequences of DM, but also to evaluate experimental approaches for the treatment of this condition [37]. As previously reported [61–63], streptozotocin-induced diabetes mellitus is characterized by body weight loss and increased blood glucose. Despite the lower final body weight in diabetic rats, blood pressure did not differ between groups. This result is in accordance with previous studies on diabetic SHR [5, 8]. Therefore, we can discard blood pressure changes participating in the cardiac alterations observed in our diabetic SHR. In experimental studies, apocynin has been used in various dosages, ranging from 4 [32] to 100 mg/kg/day [64, 65]. However, moderate apocynin doses, as administered to our rats (16 mg/kg/day), have often been used in rodents [31, 66]. In this study, as in previous reports, apocynin did not modulate body weight, glycemia levels or systemic blood pressure [31, 32, 67, 68].

In this study, diabetic cardiomyopathy was characterized by left chambers dilation, LV mass increase, and diastolic dysfunction in SHR. Myocardial functional changes in SHR-DM included impaired systolic and diastolic function, characterized by a decreased maximum tension development rate and maximum tension decline rate and increased time to peak tension at basal contraction and after all positive inotropic stimulation. Similar structural and functional cardiac alterations have been described in normotensive and hypertensive diabetic rats [5, 18, 69–71]. Cardiac function in elderly SHR was more extensively jeopardized by diabetes than in this study with younger SHR [5].

As potential mechanisms involved in cardiac dilation and dysfunction, we observed that SHR-DM had increased oxidative stress, characterized by higher myocardial lipid hydroperoxide concentration, with altered activity of anti-oxidant enzymes glutathione peroxidase and catalase. SHR-DM also present higher myocardial fibrosis, as shown by the increased myocardial hydroxyproline concentration. Hydroxyproline is the main component of the collagen molecule and can only be found at small concentrations in a limited number of other proteins. Evaluation of myocardial fibrosis through hydroxyproline is considered more precise than histological analyses [49]. Mechanical properties of myocardium are influenced by both the content and type of collagen. Fibrillar collagen types I and III are the main components of the cardiac extracellular matrix [72]. Type I collagen is associated with higher stiffness in different tissues than type III collagen [73, 74]. Alterations in collagen fiber quantities and type have often been observed in diabetic cardiomyopathy [69, 75]. Therefore, in this study, the increased collagen concentration, reduced type III collagen expression, and augmented oxidative stress may be involved in the cardiac and myocardial dysfunction observed in SHR-DM.

Apocynin administration did not change cardiac structures or function in control SHR. However, in papillary muscles, apocynin reduced developed tension at basal conditions and in post-rest contraction in SHR-APO compared to SHR. Despite lower glutathione peroxidase activity in SHR-APO, oxidative stress did not differ between SHR-APO and SHR. We can therefore conclude that apocynin impaired myocardial function independently of changes in oxidative stress. This result was unexpected as apocynin displayed beneficial effects in different experimental models of cardiac injury. In rodents with pressure overload induced by abdominal aorta banding [76] or angiotensin II infusion [77], apocynin attenuated cardiac hypertrophy, oxidative stress, cardiac fibrosis, and diastolic dysfunction. These effects were mediated at least in part through a pathway involving NADPH oxidase [77]. Also in type 4 cardiorenal syndrome [78] and chronic renal failure [79], apocynin attenuated cardiac injury. On the other hand, apocynin can exert cytotoxic effects in cell cultures [80]. One study recently showed that apocynin downregulates Akt activity in mouse embryonic stem cells [35]. Also in vascular smooth muscle cells, apocynin prevented the activation of Akt by hydrogen peroxide and by intracellular radical generator menadione [20]. The Akt signaling pathway is involved in myocyte cell surviving [81]; it is therefore possible that its inhibition contributed to the myocardial impairment seen in SHR-APO. As, to the best of our knowledge, this is the first study to evaluate the effects of apocynin on cardiac remodeling in adult SHR, we cannot compare our results with literature data.

In diabetic SHR, apocynin reduced oxidative stress, evaluated by the myocardial lipid hydroperoxide concentration, and preserved anti-oxidant enzyme activity in SHR-DM-APO compared to SHR-DM. Despite the lower oxidative stress, LV and myocardial function was not changed by apocynin, except for a lower post-rest contraction −dT/dt in SHR-DM-APO than SHR-DM. Myocardial fibrosis did not differ from SHR-DM.

Apocynin can be isolated and purified from several plant species such as *Picrorhiza kurroa*, and has been evaluated in experimental studies as an anti-oxidant agent [82–84]. It has mostly been used as an inhibitor of NADPH oxidase activity [20, 21, 82, 85]. In this study, NADPH oxidase activity and AGEs did not differ between groups. It is possible that adult SHR already have increased basal levels of NADPH oxidase activity and AGEs [86, 87] which could not be modulated by diabetes or apocynin. In fact, 3-month old diabetic SHR presented higher cardiac NADPH oxidase activity than control SHR [88]. Thus, apocynin reduced myocardial oxidative stress in SHR-DM-APO through mechanisms not involving NADPH oxidase activity. In cultured cells, apocynin acted as a ROS scavenger and not an NADPH oxidase inhibitor [20]. Apocynin can also modulate nitric oxide-dependent pathways. Apocynin has been shown to increase nitric oxide bioavailability through, at least partly, induction of nitric oxide synthase [89]. In vivo studies showed that apocynin restored normal flow mediated nitric oxide signaling in young SHR arteries [90] and increased nitric oxide synthase activity in persistent pulmonary hypertension of the newborn [91]. Additional studies are needed to clarify the effects of apocynin on cardiac remodeling in SHR with and without diabetes.

In conclusion, apocynin reduces oxidative stress through unrelated NADPH oxidase activity mechanisms and does not change ventricular or myocardial function in spontaneously hypertensive rats with type 1 diabetes. The apocynin-induced myocardial functional impairment in spontaneously hypertensive rats without diabetes shows that apocynin action during sustained chronic pressure overload needs to be clarified.

## Abbreviations

A wave: late diastolic mitral inflow velocity
AGE: advanced glycation end-products
APO: apocynin
BP: systolic blood pressure
BW: body weight
CSA: cross-sectional area
DHE: dihydroethidium
DM: diabetes mellitus
DT: peak developed tension
−dT/dt: maximum tension decline rate
+dT/dt: maximum tension development rate
E: ethidium
E’: tissue Doppler imaging of early mitral annulus diastolic velocity
E wave: early diastolic mitral inflow velocity
E/A: ratio between early (E)-to-late (A) diastolic mitral inflow
EOH: 2-hydroxyethidium
GSH: glutathione reduced
GSH-Px: glutathione peroxidase
HOP: myocardial hydroxyproline concentration
ICF: myocardial interstitial collagen fraction
Iso: isoproterenol
IVRT: isovolumetric relaxation time
LA: left atrium diameter
LH: lipid hydroperoxide
Lmax: apex of the length-tension curve
LV: left ventricle
LVDD: left ventricle diastolic diameter
LVM: left ventricle mass
MFS: midwall fractional shortening
NADPH: nicotinamide adenine dinucleotide phosphate
PP30: 30 s post-rest contraction
PWT: left ventricle diastolic posterior wall thickness
ROS: reactive oxygen species
RT: resting tension
RWT: left ventricle relative wall thickness
S’: mitral annulus systolic velocity
SHR: spontaneously hypertensive rats
SOD: superoxide dismutase
SWT: left ventricle systolic posterior wall thickness
TDI: tissue Doppler imaging
TPT: time to peak tension
[Ca^+2^]_0_: extracellular Ca^2+^ concentration
